# Computational methods for the characterization of *Apis mellifera* comb architecture

**DOI:** 10.1038/s42003-022-03328-6

**Published:** 2022-05-16

**Authors:** Christoph Bader, João Costa, Nic Lee, Rachel Smith, Ren Ri, James C. Weaver, Neri Oxman

**Affiliations:** 1 MIT Media Lab, Massachusetts Institute of Technology, Cambridge, MA 02139 USA; 2 Wyss Institute for Biologically Inspired Engineering, Harvard University, Boston, MA 02115 USA

**Keywords:** Structural biology, Computational biology and bioinformatics

## Abstract

The architecture of honey bee combs embodies a range of expressions associated with swarm intelligence, emergent behaviors, and social organization, which has drawn scientists to study them as a model of collective construction processes. Until recently, however, the development of models to characterize comb-building behavior has relied heavily on laborious manual observations and measurements. The use of high-throughput multi-scale analyses to investigate the geometric features of *Apis mellifera* comb therefore has the potential to vastly expand our understanding of comb-building processes. Inspired by this potential, here we explore connections between geometry and behavior by utilizing computational methods for the detailed examination of hives constructed within environments designed to observe how natural building rule sets respond to environmental perturbations. Using combs reconstructed from X-ray micro-computed tomography source data, we introduce a set of tools to analyze geometry and material distributions from these scans, spanning from individual cells to whole-hive-level length scales. Our results reveal relationships between cell geometry and comb morphology, enable the generalization of prior research on build direction, demonstrate the viability of our methods for isolating specific features of comb architecture, and illustrate how these results may be employed to investigate hive-level behaviors related to build-order and material distributions.

## Abstract (web summary)

Digital reconstruction of honey bee combs using X-ray micro-computed tomography provides a computational tool to analyze comb geometry and material distribution. Ranging from the individual-cell scale to the whole hive, this tool allows investigation of the relationship between comb architecture, the environment, and social behavior.

## Introduction

For centuries, the construction of combs by social insects has been endorsed as an archetype of swarm intelligence and self-organization. Hypotheses regarding the behaviors that enable comb-building range from a top-down blueprint inherited by individuals^1^ to a bottom-up emergent organization arising from the actions of agents in a collective^2^ Despite their structural complexity, models exploring these behaviors, which lead to the construction of organized comb cells^3^, construction of parallel combs^4^ or controlled distribution of brood or honey^5^ in beehives, have traditionally relied on detailed physical measurements and the direct observation of combs with the naked-eye^6,7^, which represents an incredibly labor-intensive and time-consuming process. To address these research bottlenecks, in this study, we introduce a computational toolset for investigating the morphology of comb built by the domestic honey bee, *Apis mellifera*. These methods provide novel means to characterize the process of comb building, thus furthering our understanding of the behaviors and health of a colony. To observe these social changes, we relate comb architecture to building behaviors to understand the mediations between bees and their environment.

**Fig. 1:**
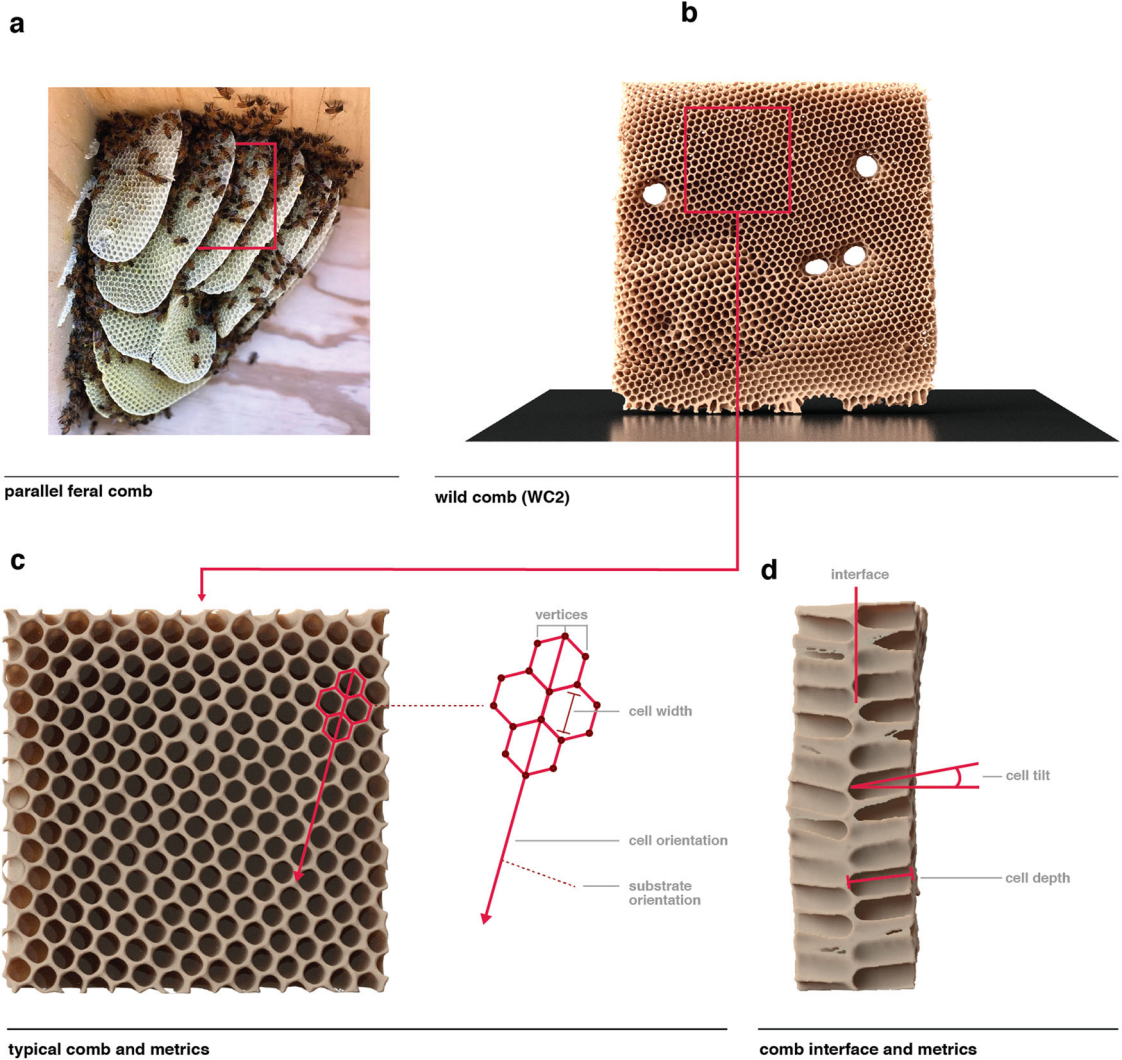
General characteristics of *A. mellifera* comb. **a** A photograph of comb being constructed by a feral bee colony in Southern California demonstrates the typical parallel lobes of comb observed in *A. mellifera* hives (image courtesy of Super Bee Rescue, Santa Barbara, California). **b** A digital reconstruction of a section of feral comb, and (**c**), an isolated section of comb cells from the digital reconstruction in (**b**), outlining the characteristics of cells including vertex locations, the measurement of cell width, and cell orientation (which has been reported to be perpendicular to substrate orientation). **d** A 90-degree rotation of (**c**) outlines the interface where the basal side of cells meet, the angle of cell tilt is measured in reference to the normal of the comb’s dominant plane, and cell depth is measured along this vector.

Typically, *A. mellifera* comb is constructed from a series of hexagonal cells with edge lengths ranging from ca. 2.25 to 2.75 mm^8^, edge thicknesses of ca. 0.75 mm^9^, and average depths ranging from ca. 10–11 mm^10^. This cell size is governed by the body size of worker bees, which have an average radius of ca. 4 mm^11^. Comb cells are built at an average angle of 13 degrees from an interface where the basal sides of two cells meet, and while it has been proposed that this angle helps better retain honey within the cell interiors, recent evidence suggest that it may also provide additional structural reinforcement for the growing comb^12^. Honey bees build parallel sheets of cells in this fashion, with an average center-to-center spacing of ca. 7 to 10 mm^13^. Comb cells may be filled with either honey to form honeycomb or larvae to form brood comb, and the quantity of comb within a hive is thus directly proportional to the hive’s need to produce young and store food. To illustrate these specific features, Fig. 1 provides a series of progressively magnified views of a feral hive. Consisting of a series of approximately parallel lobes (Fig. 1a), the combs are constructed upon an underlying substrate. Within a planar array (Fig. 1b) of predominantly uniform hexagonal unit cells (Fig. 1c), we can define the following geometric features (Fig. 1c): (1) the cell width, (2) the unit-cell vertices (the corners of a cell where three edges meet), and (3) the cell orientation (the vector that can be drawn continuously from the highest to the lowest vertex of a cell); and from a sectioned view of the comb (Fig. 1d), (4) the cell depth, and (5) the inclination angle of each cell.

These parallel sheets and packed hexagonal cells are reported to approach their theoretical maximum packing density, while still allowing for efficient bee navigation within the hive, a phenomenon described by Darchen’s rule of *parallelism*^14^. However, deviation from this pattern can be observed under specific circumstances. For example, feral honey bee comb frequently contains roughly parallel (yet non-vertical) regions that follow the contours of the structures on which they are built. This effect has also been observed under experimental conditions, where parallel comb construction could be interrupted by obstructing the targeted build direction of the hive with a smooth sheet of glass, and the subsequent redirection of comb building to form a curved sheet^8^. Since these cell-level deviations inherently lead to the creation of structures that are sub-optimal for the storage of larvae and honey, they present an opportunity to better understand the ways in which hives may adapt comb architecture in response to environmental stimuli. To help address these questions, here we introduce a reproducible experimental research platform for the controlled generation of geometrically complex (non-parallel) comb architectures through the manual rotation of hives during the comb-building process.

While historically, measuring the geometric characteristics of honey bee comb was performed by hand, more recent efforts have employed machine-learning methods to automate the identification of cell types in photographs of combs^15^, or micro-computed tomography (µCT) data to characterize three-dimensional (3D) material distributions within individual cells^16^. While these small-scale measurements have provided some intriguing insights into cell structural diversity and their stages of formation, the use of high-resolution volumetric data sets to study the geometric features of *A. mellifera* combs at the whole-hive scale has remained largely unexplored. We propose that such large-scale 3D data sets could thus provide a valuable resource for investigating multi-scale aspects of comb architecture, and when combined with computational geometry methods, enable the automated high-throughput characterization and quantification of features, which have previously been studied only through manual approaches, to provide insights into the comb construction process.

In the following sections, we present a three-part framework for developing toolsets and achieving rapid digital characterization of comb architecture. First, we use micro-computed X-ray tomography (micro-CT) for the generation of fully interactive 3D comb models, providing a baseline for the investigation of comb properties, including geometry and material density, as well as their spatial variations within the hive. Second, we introduce toolsets and methods adapted from computational geometry for the shape interrogation of natural combs. Lastly, we use these tools in combination to elucidate rules that govern comb-building behaviors and, ultimately, the hive’s final structure. Through these methods, we enable the validation of previously proposed models and observe new behavioral changes manifested in the relations between geometric expressions and comb construction.

## Results

**Fig. 2:**
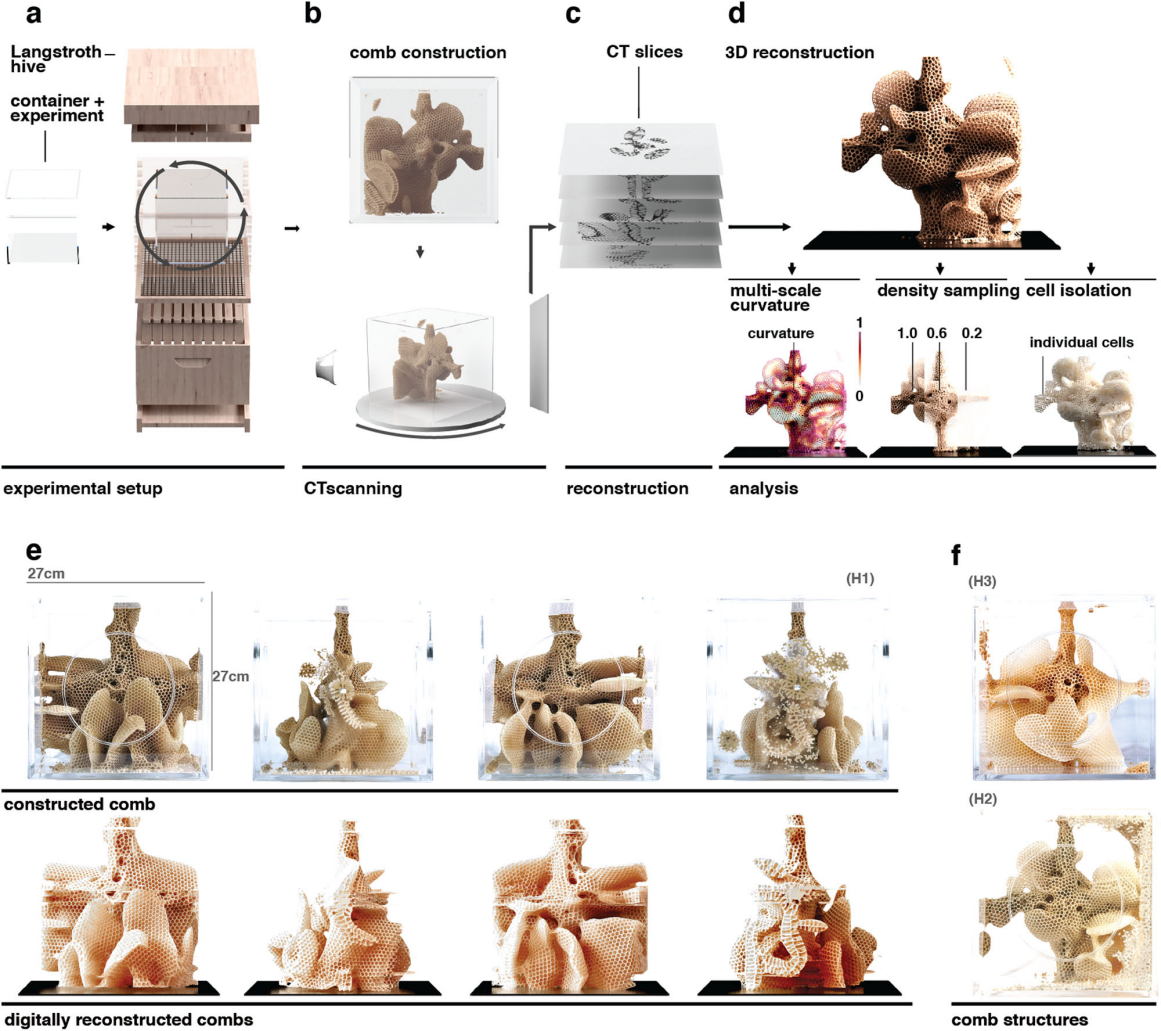
Hive experimental setup and data digitization workflow. **a** In our experimental approach, combs were constructed by *A. mellifera* colonies inside experimental acrylic cubes that were integrated into standard Langstroth hives. Periodic rotations applied to the acrylic cubes during hive construction induced changes in the hive’s build direction, resulting in combs with atypical protrusions and highly curved geometries. **b** After the comb construction process was complete, the acrylic cubes were removed from the hives and scanned using a commercial micro-CT imaging system. **c** For each cube, 2000 16-bit z slices at a resolution of 2000 × 2000 pixels were generated. **d** The slices were reassembled into a volumetric data set and stored as OpenVDB files for further analysis. **e** Photographs (upper) and digital reconstructions (lower) produced by the described workflow are shown for Hive 1. **f** Photographs showing two additional replicate experimental cubes (Hive 2 and Hive 3) produced and digitized with this workflow.

In order to examine the efficacy of the developed computational analysis tools, it was desirable to obtain a honey bee comb with complex architectures. In the present study, data were obtained from *A. mellifera* hives that were cultivated in Cambridge, Massachusetts between February and October 2020. Once the colonies established a stable population, experimental environments, each consisting of an acrylic cube measuring either 15 cm × 15 cm × 15 cm or 27 cm × 27 cm × 27 cm, were incorporated into their existing commercially available Langstroth hives (Fig. 2a). By rotating these environments in sequences described in the Methods section, perturbations in comb-building behavior were induced that resulted in the creation of hives with highly curved combs protruding in many different directions (Fig. 2b–f).

### Data representations for geometric analysis

**Fig. 3:**
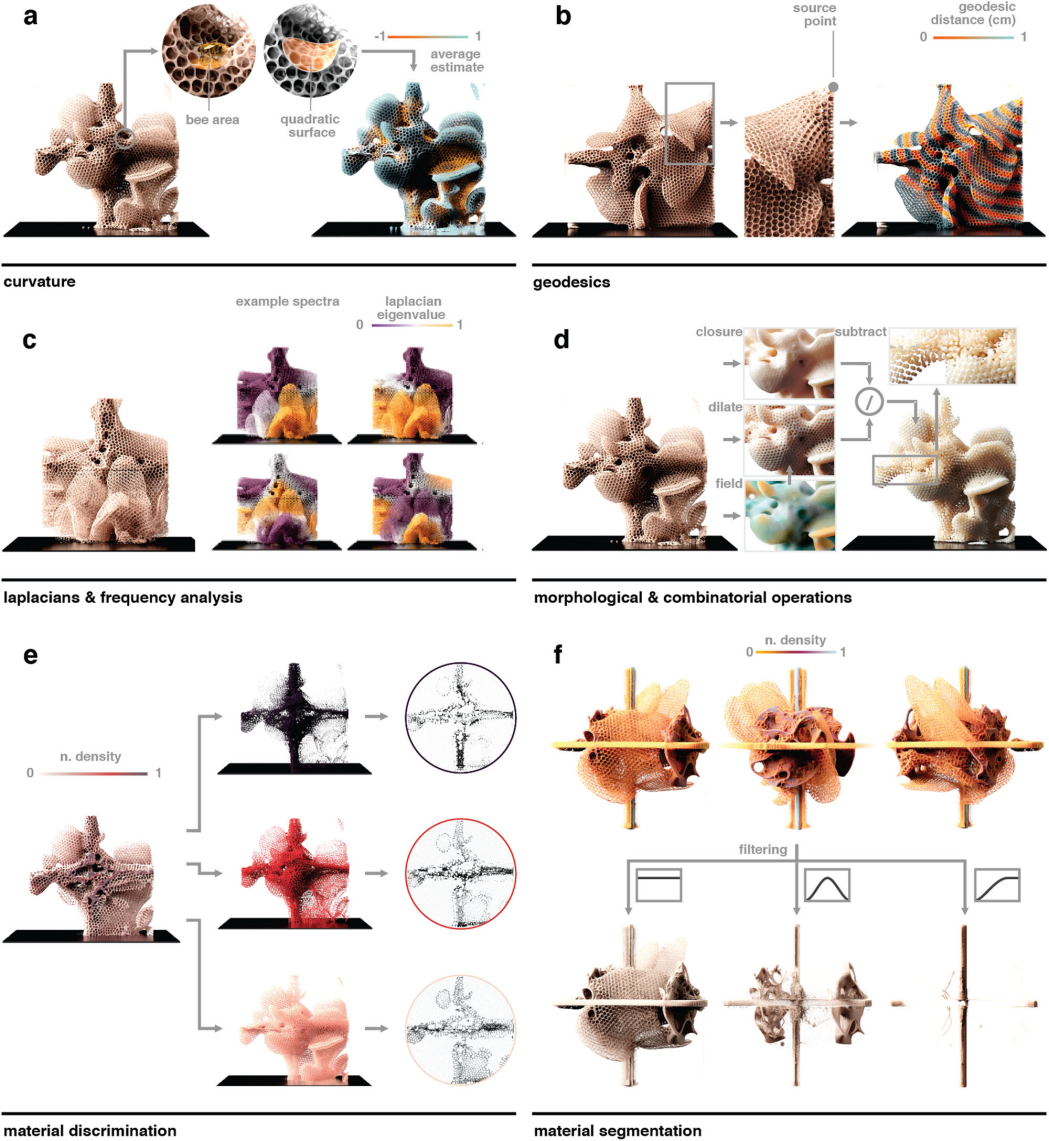
Primary computational geometry tools used to analyze comb architectures. **a** To compute a Gaussian curvature estimation across a digital comb topography, at every point of the comb structure, a radius (*r* = 15 mm) corresponding to the size of an *A. mellifera* worker was used to sample neighboring positions to which a quadratic surface was fitted. The quadratic form was then used to compute the curvature analytically. **b** To plot a geodesic distance from a source point, the geodesic distance was computed on the surface of the shown comb structure, and 1-cm periods were plotted to show equidistant propagation from the selected point. **c** Different harmonic bases of the Laplace operator are shown with color values denoting the per-vertex amplitudes. Together with the eigenvalues of the Laplace operator, these are commonly employed as shape descriptors. **d** Morphological operations of dilation and closing were used in combination with combinatorial operators, such as subtraction and intersection, to extract cells and tunnels from comb architectures. **e** Normalized attenuation contrast values from X-ray data were used to discriminate different materials within the combs; i.e., bee-deposited wax, manually applied wax foundation, and 3D-printed polymer or acrylic support structures. **f** Thus, substructures of different density ranges could be selectively filtered for and visualized in the digital environment. *A. mellifera* constructed combs are displayed yellow, 3D-printed parts are shown in magenta (density = 1.19 g/cm³), and steel rods are displayed in cyan.

While the volumetric data obtained from micro-CT scans of our experimental hives were originally stored as OpenVDB files, we utilized three different representations to perform our geometric analysis to make use of their respective advantages. To begin, we assumed that the comb was a solid $S$. The first representation was a volumetric representation of the data set composed of a set of voxels $V={\left\{{v}_{i}\right\}}_{0\le i < {2000}^{3}}$ with positions $p({v}_{i})\in {R}^{3}$ within a regular grid. Voxel values $0\le i({v}_{i})\le 1$ represented the normalized density values of the respective materials at their spatial voxel positions. However, for methods that do not examine differences in material density, it can be advantageous to treat the volume as though its density were constant, which was used for calculating a signed distance field (SDF)^17^, as described below. Here, the comb shape was represented by a signed distance function ${d}_{S}\left({v}_{i}\right)={{{\rm{sign}}}}({v}_{i}){{\inf }}_{x\in \partial S}\Vert x-p({v}_{i})\Vert$, where ${{{\rm{sign}}}}\left({v}_{i}\right)=\left\{-1{if}{v}_{i}\in S,1{if}{v}_{i}\in S\right\}$ was evaluated at each voxel (a value of −1 denotes a point inside the volume, while a value of 1 denotes one that is outside). The third representation is the discretization of $\partial S$ as a triangle mesh representing the boundary of our comb solid $S$. In this case, a triangle mesh represents a collection of vertices $W=\{{w}_{j}\}$, edges $E=\{{e}_{k}\},{e}_{k}\in W\times W$, and triangles $T=\{{t}_{q}\},{t}_{q}\in E\times E\times E$ with associated attributes, such as position. In the following section, we introduce a set of computational geometry tools that were used to analyze our different honeycomb architectures (Fig. 3).

### Development of a Gaussian curvature tool

Since the comb’s curvature may indicate a deviation from typical parallel construction behavior, the calculation of curvature was a primary goal of these analyses. For example, Gaussian curvature indicates when a structure is doubly curved, or curved in multiple directions at a specific location, while mean curvature is a signed (positive or negative) value that indicates curvature in two principal directions perpendicular to the surface normal. The Gaussian curvature $K={\kappa }_{1}{\kappa }_{2}$ is the square of the geometric mean of the principal curvatures ${\kappa }_{1}$ and ${\kappa }_{2}$, and mean curvature $H=\frac{{\kappa }_{1}+{\kappa }_{2}}{2}$ is the arithmetic mean of ${\kappa }_{1}$ and ${\kappa }_{2}$. These principal curvatures describe the maximum ${\kappa }_{1}$ and minimum ${\kappa }_{2}$ curvature at every point on $\partial S$. In our analysis, curvature was used to assess the local deviation from typically parallel and planar comb building. Intuitively, a local positive Gaussian curvature corresponds to a local paraboloid surface, whereas a negative Gaussian curvature corresponds to a local hyperboloid surface. We estimated curvatures through the local quadratic surface fitting, and at every point of $\partial S$, we sampled the local neighborhood in a 15-mm radius, relating the sampling region to the average size of an *A. mellifera* individual. Since the hive interior is dark, worker bees constructing comb are only able to assess hive geometry based on tactile signals from their immediate surroundings, and so sampling at a region of this size provides an assessment of curvature at the level that can be sensed by individual bees during comb construction. From this local sampling of the comb structure, we constructed a quadratic form $f\left(x,y\right)=a{x}^{2}+b{y}^{2}+{cxy}+{dx}+{ey}$, with which both the Gaussian and mean curvatures can be estimated analytically by computing the eigenvalues of the shape operator^18^. The estimated values were then locally averaged, an example of which is shown in Fig. 3a.

### Development of a geodesic distance measuring tool

To understand comb architecture from the perspective of a honey bee, it is important to consider that bee movement within a hive’s interior is limited to travel across the comb surface and the walls of the container, since the confined space and lack of light limits bees from flying within the hive. To provide a sense of distance as it would be experienced by a bee traversing the comb surface, geodesic distance is a useful parameter to consider since it describes the shortest distance from a point in the domain, on $\partial S$ or within $S$, to any other point in the domain, on $\partial S$ or within $S$, respectively, and can be defined through the solution of the eikonal equation $\left|\nabla u(x)\right|=\frac{1}{s(x)}$ [Eq. 1]. Several algorithms have been developed to compute these distances, including the heat and fast marching methods^19^. Distance computations can be weighted by a metric $s(x)$, which is a field over the surface or solid that can be used to model the difficulty of traveling through a specific region. In the present study, the volume’s density value was used as the weight metric $s(x)$ so as to obtain the distance along the object’s surface. Given the geodesic distance field from a source to every point in the domain, the gradient of this field can be used to construct the shortest paths within the domain (Fig. 3b).

### Development of Laplacian and frequency analysis tools

To understand comb architecture at a scale larger than an individual bee, frequency analysis can be used as a tool to isolate and segment individual lobes or sections of comb. Such segmentation can be accomplished through the analysis of the Laplace operator eigenvalues. The Laplace operator $\triangle$ is a linear operator that can be applied to the Cartesian xyz position of a point in a mesh representation of the object in order to evaluate the attribute’s local deviation from its mean value, and is commonly employed for investigating diffusion flow, geodesic computations, and interpolation^20^. Since the Laplace operator is linear and positive semidefinite, its eigenvectors comprise an orthogonal basis, which is the manifold harmonic basis for the attributes on our domain. The projection of an attribute to this basis results in a spectral representation that is often used in its frequency manipulation. Basic functions of this basis are shown in Fig. 3c, with color values denoting the per-vertex amplitudes.

### Development of morphological and Boolean operation tools

To accomplish tasks such as quantifying the number of cells or analyzing their tilt, it can be useful to combine operations that isolate the negative space within the analyzed volume. Morphological operators comprise those that modify the volume’s density values by expansion or erosion, while Boolean operators consist of those that combine two inputs. Given a ball $B=\left\{{b|d}(b,({{\mathrm{0,0}}}))\,\le r\right\}$ of radius $r$ with a distance function $d$, the morphological operators of dilation can be defined as $V\oplus B=\{{v|}{{{\rm{sign}}}}\,\left(v\right)* d(v)\le r\}$ [Eq. 2] and erosion as $V\ominus B=\{{v|}{{{\rm{sign}}}}\,\left(v\right)* d(v)\le -r\}$ [Eq. 3]. The composition of these operators yields morphological closing $(V\oplus B)\ominus B$ and opening $(V\ominus B)\oplus B$ operations, which can in turn be combined to yield algorithms for closing volumetric objects^21^. We used morphological operators on the SDF representation to extract geometrical features, such as the cells and tunnels of the hives^22^. By pairing morphological operators with Boolean operators, such as subtraction or intersection of volumes, we were able to extract these features from our combs, as shown in Fig. 3d.

### Development of a density discrimination tool

We also explored the use of variability in attenuation contrast values from micro-CT scans to characterize material distributions within the comb (Fig. 3e). Due to the elemental similarity of organic materials that are incorporated into the growing hive, variability in attenuation contrast is assumed to correlate to physical density (which can vary as a function of water or lipid content or intrinsic porosity). For example, new beeswax has a translucent-white color and a typical density^23^ of 0.958–0.970 g/cm^3^. Honey bees also produce propolis, at a measured density of 1.16 g/cm^3^, through the combination of saliva, wax, and plant-produced resins^24^. In addition to the potential identification of site-specific material deposition during hive construction, the attenuation contrast values from the micro-CT scans can also be used to separate comb from manually applied foundational wax and support structures of various materials, as shown in Fig. 3f. Since our micro-CT scans were taken from experiments in which *A. mellifera* constructed combs (yellow) on 3D-printed parts (magenta, density = 1.19 g/cm³) held by steel rods (cyan), material discrimination allowed analysis of constructed combs independent of other structures in the experimental setup.

### Application of the developed tools

We used the computational geometry tools described in the previous sections to analyze comb architectures, validate common assumptions about comb construction, and make connections between bee behavior and comb geometry. The approaches presented for characterizing comb volumetric data sets include (1) curvature-based analysis to analyze the distortion and deformation of cell geometries, (2) geodesic distance analysis to examine build direction, and (3) algorithms for isolating and viewing the comb interface and cell tilt (see Fig. 1d).

Wild combs are typically constructed as double-sided sheets of tessellated hexagonal cells. Comb construction is often guided by the rule of parallelism, first hypothesized by Darchen^14^, who observed that while bees may start to build clusters at different attachment sites, they modify their construction to keep a reasonably equal and parallel space between adjacent combs. Further, it has been previously proposed that cavity-nesting honey bees, such as *A. mellifera*, tend to establish a build direction in line with the force of gravity^10^. The combination of the unit-cell geometry and their parallel sheet construction has been previously proposed to represent optimal building material use to maximize honey and brood storage space^25^. In our current study, rotating the hives during the building process and providing variations in substrate geometry produced perturbations to the bees’ building environment that resulted in parallelism being disrupted and bees adjusting combs through torsion and irregular cell-building behavior. These perturbations induced curvature into the comb similar to that reported by Huber in studies where glass sheets were used to obstruct the intended direction of comb building^8^.

**Fig. 4:**
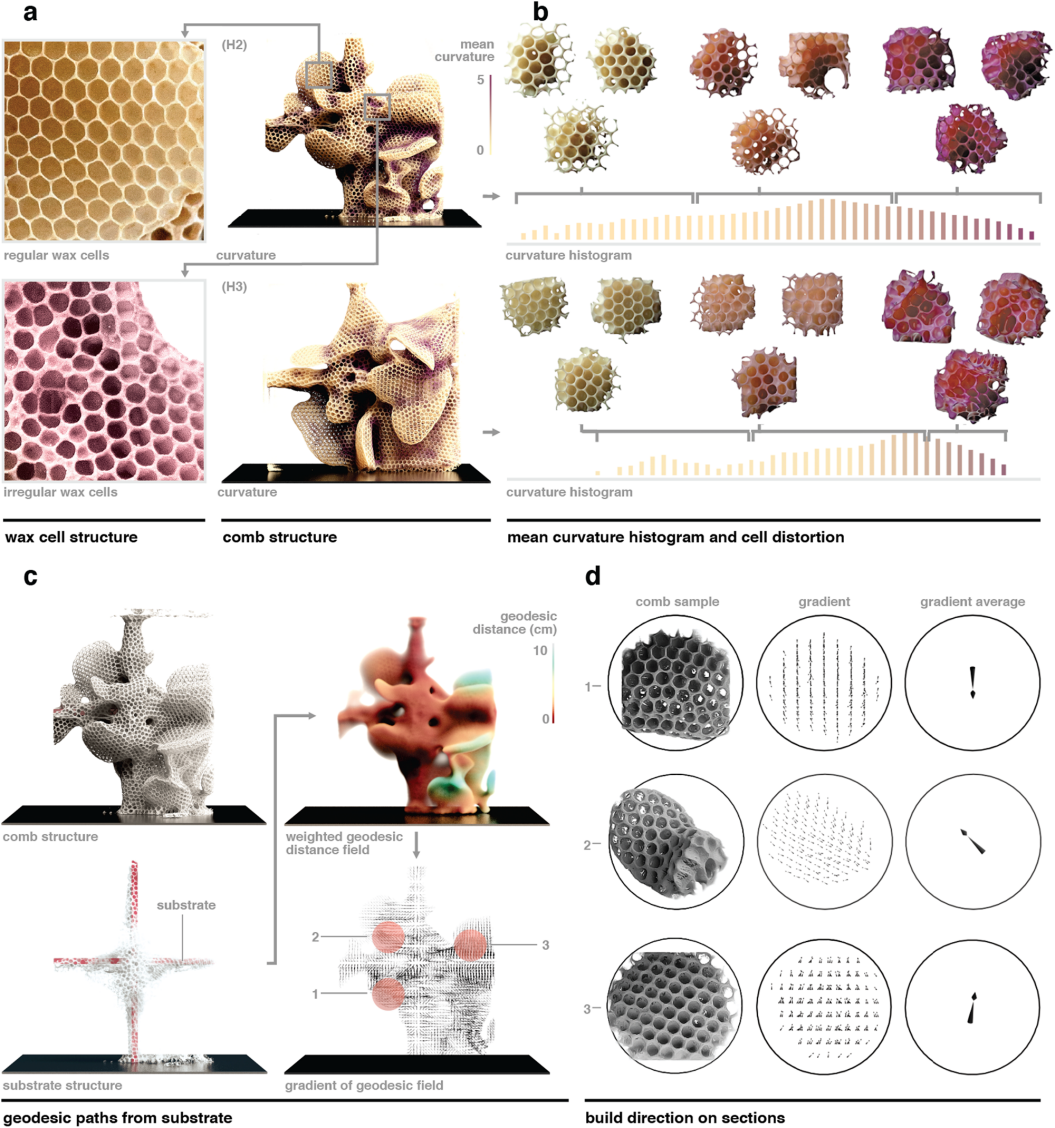
Digitally driven observations of cell regularity and build direction. **a** Visually identified examples of regular (upper) and irregular (lower) cells within a comb digital reconstruction. **b** The relationship between comb curvature and irregular cell construction is analyzed for the comb architectures of two experimental cubes (Hives 2 and 3). Using the established Gaussian curvature tool, the combs are color-coded according to their principal curvature ${\kappa }_{1}$, distinguishing high curvatures (violet) and low curvatures (yellow). Curvature histograms of ${\kappa }_{1}$ are shown for each of the hives, displaying the distribution of curvature across the combs, and their corresponding color-coded images illustrate the extent of local cell distortion. **c** Comb build direction is compared to the gradient of the distance field from the initial underlying substrate. For the Hive 2 comb, the geodesic distance field is color-coded (red to blue) by increasing distance from the initial underlying substrate (top-right), and its gradient is shown as a vector field (bottom-right). Three sample regions of interest were selected for further analysis. **d** For each sample region, visualized (left), the gradients of the geodesic field over the region are displayed (middle), and the average of these gradients is calculated (right). The resulting vector corresponds to the bees’ build direction, as identified by Pratt’s method using one vertex of each cell oriented such that it points toward the substrate.

Regarding cell shape, Fig. 4a–c demonstrates the use of computational geometry tools to identify an association between irregular cells and negative comb curvature. Here, we used the curvature estimation method, as introduced in the Methods section, approximating a quadratic surface the size of a bee at every point over the surface, to evaluate principal curvatures. Regions of irregular comb building (Fig. 4a), potentially resulting from the geometric accumulation of locally deformed cells, can be correlated to negative principal curvatures of ${\kappa }_{1}$ and ${\kappa }_{2}$, while regular comb building is related to zero or positive curvatures. Figure 4b shows high principal curvature values in violet and low values in yellow. Curvature fields were computed for two comb structures (Hives 2 and 3). In Fig. 4c, we show curvature histograms of the comb architectures and randomly sampled regions of cells for each range of curvature, exhibiting a relation between a high negative curvature and more irregular comb building.

In regard to cell orientation, it has been shown previously that cell orientation typically depends on substrate orientation^6^, and cells are built from a substrate with one vertex of each cell oriented such that it points toward the substrate. We validated and generalized this relationship by examining whether cell orientation is aligned with the gradient of the weighted geodesic distance from the substrate. In the process shown in Fig. 4d, we computed the density-weighted geodesic distances in our micro-CT scan data from the acrylic cross substrate using the geodesic distance measuring tool. Subsequently, we evaluated the gradient of this field using finite differencing. To determine local cell orientation, we compared the average of the gradient field for a region to the regional comb geometry from the micro-CT scan data, as shown in Fig. 4e. From these analyses, we observed that the average of the gradient of the weighted geodesic distance from the substrate aligns with Pratt’s analysis of the bees’ build direction, as determined by the cells’ vertex orientation.

**Fig. 5:**
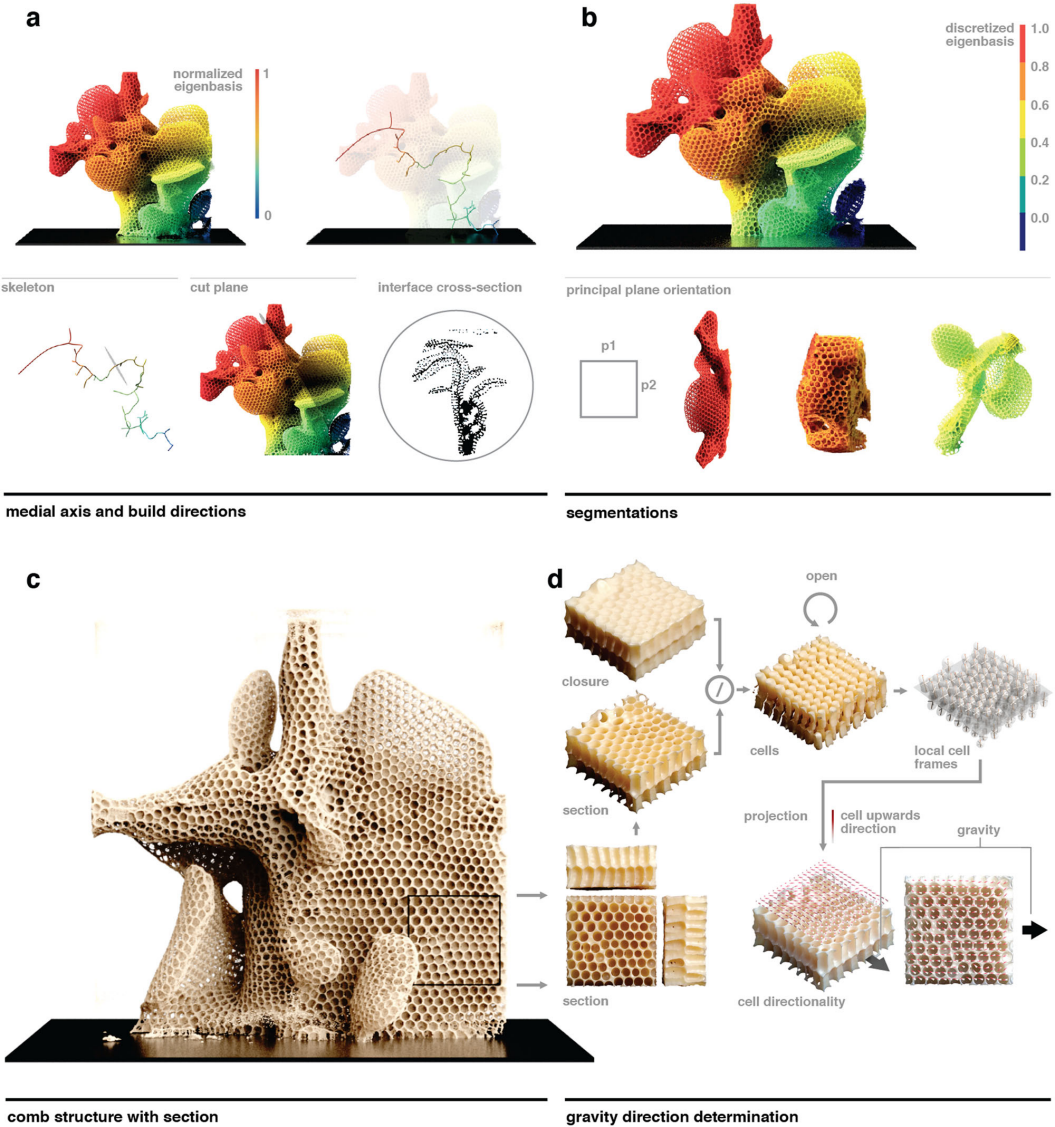
Development of global descriptors of comb and building processes. **a** Curve skeletons and cross-sections are shown, dividing the combs at their medial plane. These cross-sections bisect cells along with the interface so that their depth, thickness, and pitch can be measured at any point. **b** Comb regions that share similar principal directionalities are shown. **c** By combining established knowledge of *A. mellifera* building behavior and computational analysis of structure, we demonstrate that the gravitational direction at the time of construction can be reconstructed for a selected region of comb. **d** For the selected comb region, morphological and combinatorial operators were used to extract individual cells, by subtracting the section itself from the closure of the section and then separating each cell by iterative opening operations. Once the internal volumes of individual cells were obtained, we computed an orthonormal basis for each cell using principal component analysis. For this comb selection, the principal axis of the orthonormal frame was almost orthogonal to the medial plane of the section and was projected onto the medial plane to reveal the upward tilt that bees typically build opposing the sensed direction of gravity.

The comb interface (Fig. 1d) can be described as the zone where the basal region of comb cells come together, or where the base of each mirrored cell sheet contacts one another in a free-hanging lobe (Fig. 1a). In the parallel comb, this interface serves as the median plane along which rows of cells are tessellated. However, in a highly curved comb, observing this interface in isolation is nontrivial. In Fig. 5a, b, the segmentation of combs was demonstrated using the previously introduced Laplace operator described in Fig. 2c. Segmentation was based on approximately planar regions of comb using the smallest nonzero eigenvector of the Laplace operator, the Fiedler vector^26^. Typically, such segmentation is applied to structures with more clearly segmented architecture. For example, Fiedler vector segmentation of the human body isolates the appendages, torso, and head. We found that in the case of such atypical comb architectures, Fiedler segmentation was capable of isolating lobes of comb which are approximately planar.

From the Fiedler segmentation, isocontours were constructed and the centroid of each contour was linked using Prim’s algorithm^27^ to construct a curve skeleton^28^. This curve skeleton provides a minimum-spanning tree that connects each region of the Fiedler segmentation. The cross-sections of the volumetric data-oriented to the curve skeleton’s tangent indicate that the comb’s interface falls in line with the curve skeleton’s normal. These cross-sections bisect cells along with the interface so that cell depth, thickness, and pitch can be measured at any point. In some cases, the selected plane bisects more than one of the comb’s segmented regions. In this case, volumetric data from the segmented region corresponding to that point’s isocontour displayed a perpendicular view of the comb’s interface, but data from the other regions may not.

Fiedler segmentation was also used to isolate comb regions that share similar principal directionalities. Comb segments with eigenvector values within a small range were selected from the volumetric data of the hive. The first two principal components of the isolated voxels were derived to provide a plane that represents the section’s orientation. The first component was found to align generally with the comb’s build direction within freeform protrusions. In hives with a greater variance in comb curvature, finer segmentation may be needed to isolate regions containing similar build directions. These results demonstrate that the Fiedler vector may be a useful tool for isolating approximately planar regions of comb and for reliably examining the comb interface in the highly curved comb.

By combining established knowledge of *A. mellifera* building behavior and computational analyses of the resulting structures, we have demonstrated that the cell orientation and therefore the build order can be reconstructed from micro-CT scan data using our morphological operators. While honey-holding cells built by bees exhibit an upward tilt from a median plane toward gravity^29^, in examples such as the perturbed comb shown in Fig. 5c, or in the analysis of wild combs that have incurred large-scale structural damage at some point in their history, we can see that determining this direction is nontrivial. To determine the direction of gravity for a comb section in such cases, in Fig. 5d, we computed the combinatorial difference between the closure with a 10-mm radius and the section itself. We then iteratively applied a morphological opening operation to the resulting SDF until none of the cells were connected to any other cell. For the resulting cell volume, we computed a principal frame using principal component analysis. The *z* axis of this orthogonal basis aligns with the principal cell orientation and points away from the median plane, whereas the *y*- and *x* axes are almost orthogonal to it. The projection of the z-axis onto the median plane reveals the tilt of individual cells and, thus, the direction of gravity.

## Discussion

**Fig. 6:**
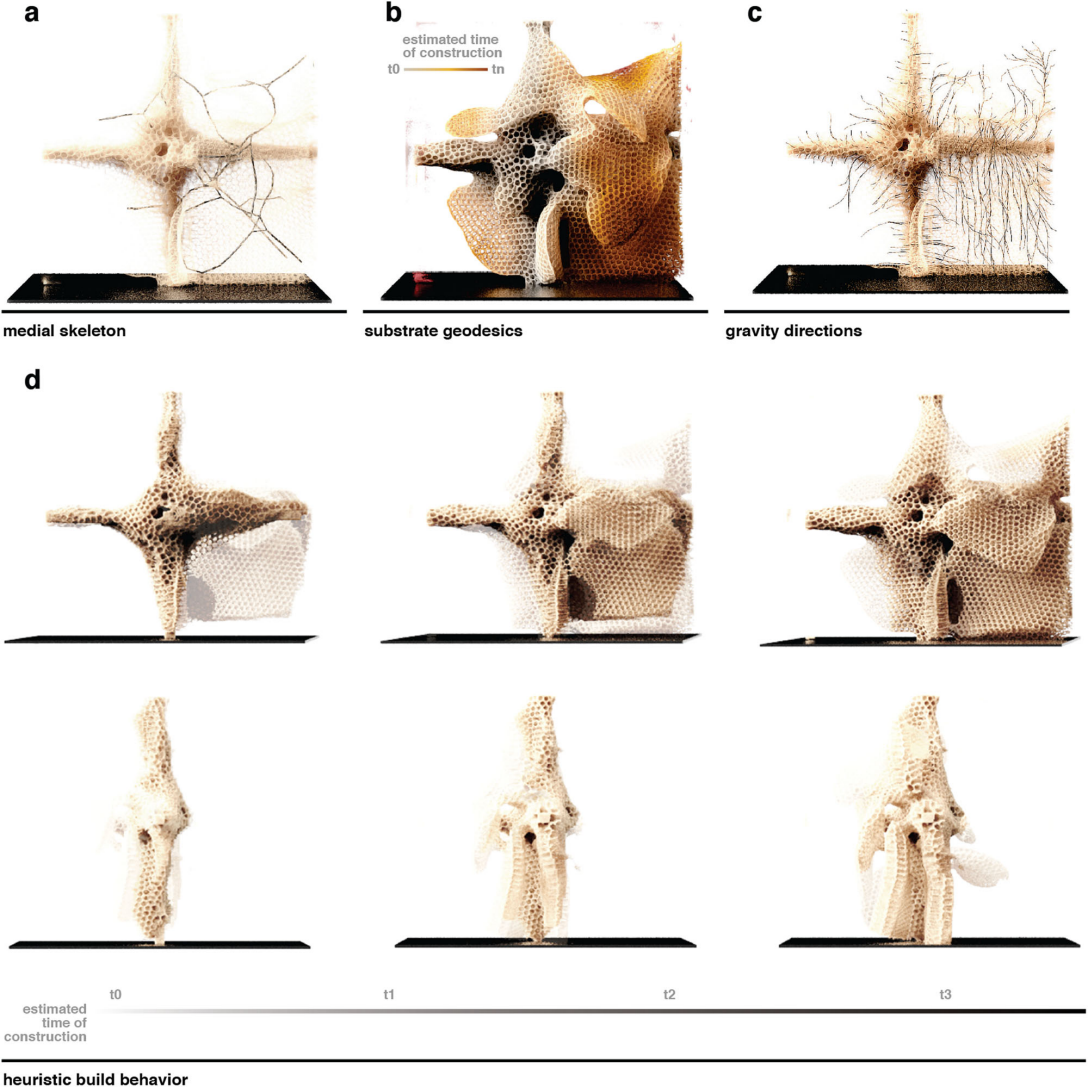
Simulation of comb construction predicated from geometric measurements. An example prediction of a comb’s construction and morphological evolution over time, using geometric data gathered by determining (**a**) the medial axis, (**b**) the geodesic field from the substrate, and (**c**) the gravity vector of each cell. For a given digital comb, we moved back along the direction of gravity and the build direction until a branch in the medial axis was detected. At this branch, we reevaluated the gravity direction and build direction and moved back until another branch was reached, and so on. From these measurements, we can generate a construction timeline (**d**), where the process was run in the reverse direction, showing the morphological changes of the combs over time, guided by the combination of gravity, substrate, and the bees’ own agency. For each timepoint, the next constructed comb section is depicted with reduced opacity.

**Fig. 7:**
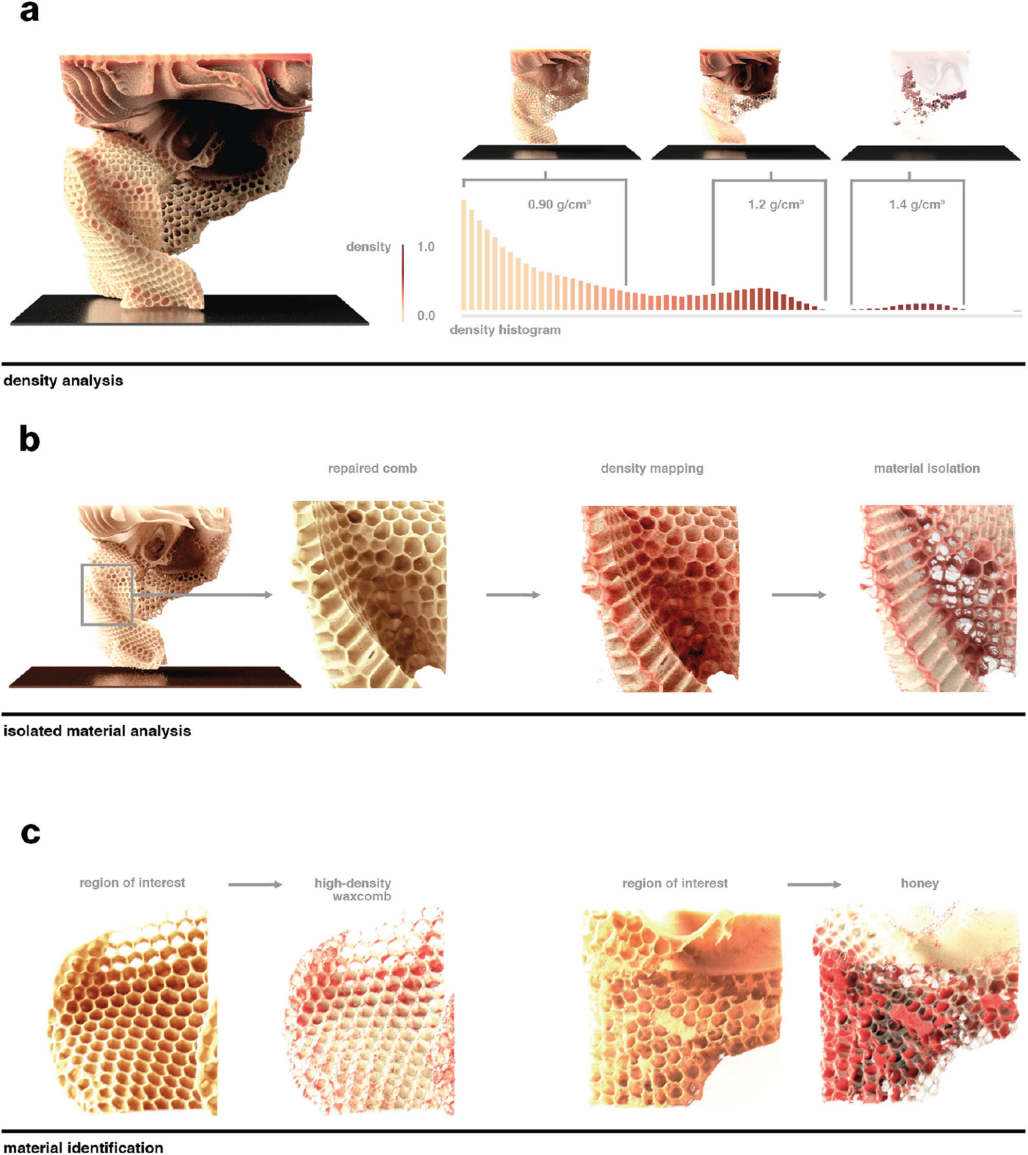
Attenuation contrast-based filtering and identification of specific material distributions. **a** Density analysis of a scanned comb (Hive F, Fig. S2) was applied to measure specific materials within a comb built on a 3D-printed substrate. The measured density values from the micro-CT scan data can be extrapolated to physical density measurements by identifying materials with measured densities within the scan. **b** Once real densities have been mapped, an isolated region of the hive where the broken comb was repaired by the colony was isolated and examined, suggesting that a higher-density wax was allocated to the fractured region and the comb’s interface. **c** This method was used to examine distributions of low- and high-density wax throughout the hive as well as to measure the volume of honey in capped cells.

As an application of the described results, we demonstrate how behaviors such as the order of comb building and the deposition of specific materials can be inferred. We propose that these methods will be most impactful when applied to honey bee behavioral studies validated by empirical measurements, and Figs. 6 and 7 illustrate how these methods may be used to examine specific behaviors within a hive.

Figure 6 shows how we reconstructed the comb build behavior and morphological evolution from the medial axis, the geodesic field from the substrate, and the gravity vector of each cell. For a given comb, starting from the endpoints of our medial axis tree, we moved back along the geodesic build direction until a branch was detected in the medial axis. At this branch, we reevaluated the gravity direction and build direction and moved back until another branch was reached, and so on. This approach provided a heuristic algorithm for tracing the comb build order.

Similarly, the distributions of specific materials within the built comb can be examined through the application of the described density-filtering mechanisms. It has been demonstrated previously that attenuation values from CT data can be used to approximate the physical density of organic materials^30^. It should be noted that this method provides only an estimate of physical density, which can be impacted by factors such as the orientation of the comb within the scanner will have an impact on attenuation contrast. To first validate our measurements, the attenuation contrast data from the micro-CT scans was fit to real-world measurements of materials with consistent densities, such as a plastic substrate and honey. From these points, the estimated density of the whole hive was then extrapolated so that materials could be examined in isolation. For example, Fig. 7 demonstrates the isolation of comb and honey in a hive built upon a 3D-printed substrate, and the extrapolated density of 0.90 to 0.96 g/cm^3^ aligns well with values reported in the previous studies^31^. Honey, which has an appreciably higher physical density than wax at 1.4 g/cm^3^, was isolated using this method to determine that 9.3 ml of honey was contained within the sample’s cells (Fig. 7).

The data from this method also suggests that smaller variations in the physical density of wax can exist throughout the comb. In this specific example (Hive F, Fig. S2), during the course of its construction, the comb was inadvertently fractured and was subsequently rebuilt by the colony. Density analysis of this damaged region suggests the presence of an atypically higher-density bridging comb—1.0 to 1.1 g/cm^3^—reconnecting the fractured segment. While these estimated variations in density are relatively small, and as such, are subject to potentially larger errors when calculating these values from attenuation contrast, the highly localized and consistent distribution of the apparently higher-density material suggests that bees may deliberately vary the physical density of comb to suit specific structural needs.

While the methods described in the present study were designed and applied for the analysis of *A. mellifera* combs, they are broadly extensible and can be applied to the structures constructed by related species. In particular, similar analyses on the combs of *Apis dorsata*^32^ or *Tetragonula carbonaria*^33^ may provide a valuable comparison between the morphological and material strategies for comb-building social insects in specific environments. Such a comparison may further the understanding of corbiculate bee phylogeny^34^ and contribute to a broader discussion regarding the evolution of eusocial behavior in such insects. Additionally, comparisons may be drawn between the structure and material distributions within domestic hives (Hives 1–3) and wild or feral colonies. To help facilitate these comparisons, micro-CT-scanned data sets of wild and feral comb sections are included in the Supplementary Information and are outlined in Fig. S3.

The application of the described methods to species that exhibit similar structural morphologies using vastly different materials would also be equally valuable. Paper wasps are an excellent example, especially since hexagonal cell-building behavior is believed to have evolved independently in corbiculate bees and paper wasps^35^. Such a comparative analysis may ascertain whether morphologies related to the alignment of hexagonal cells, the construction of the interface, and the alignment of comb segments are analogous across species and materials, potentially indicating behavioral similarities.

Despite the usefulness of the methods described in the present study, a challenge to the widespread adoption of these analytical tools is the requirement of access to both well-preserved samples of combs and equipment for the acquisition of micro-CT data sets, which can be prohibitively expensive. While this may be the case, it is possible that portable and lower-cost methods of 3D data acquisition, such as photogrammetry or laser scanning, may enable at least the surface-level analyses described here (cell orientation, curvature measurement) in an accessible manner. To facilitate research more broadly, however, it would be advantageous to initiate a large-scale effort to collect, preserve, and scan samples of combs from wild and domestic hives across multiple species. This data, if disseminated through an accessible database and coupled with the methods described here, may provide additional data points in the research on these commercially important species. Ideally, such data would include measurements of external environmental parameters, colony population density, survival rate, and hive temperature to examine hypotheses related to pollinator health. Hence, we invite the entomological research community to apply the methods described here broadly and across species, in hopes that such discourse will provide a deeper understanding of topics, ranging from emergent organization to large-scale ecological health.

The workflows and analytical tools presented here enable, for the first time, a robust analysis of corbiculate beehives. While previous methods have relied on partial examination of isolated comb structures, manual systems of measurement, or naked-eye observations, the methods described here represent a high-throughput process that is well suited for automation and comparative analysis. By applying these methods to several complex *A. mellifera* hives, we reexamined various hypotheses related to the morphology and construction of honey bee combs.

Specifically, we demonstrated that the orientation of hexagonal cells, which has previously been shown to be dependent on the orientation of the comb’s substrate, maintains this relationship along the geodesic path from the cell to the substrate. We further provided methods by which the geometric characteristics of a comb can be quantified—for example, its curvature at various scales, the size and distribution of cells, and variations in material density. We also reconstructed how gravity and rotation influence the build behavior of bees. Lastly, we outlined computational techniques by which specific anatomical characteristics, such as irregular cells or the comb’s interface, can be isolated. Although these are just the beginnings of elucidating a dialog between geometry and behavior, the multi-scale analysis of comb geometry described here helps lay the groundwork for more detailed studies investigating the environmentally directed behavioral adaptations of honey bees.

The aim of the present study was to illustrate how these methods can be applied to deepen our understanding of honey bee behavior, particularly in relation to swarm intelligence and collective strategies for building and construction. Beyond this goal, these methods may serve to augment the study of honey bee health at a time when their preservation is more vital than ever. We hope that these methods provide the bee research community with a novel toolkit that can be used to study, understand, and protect these critical pollinators.

## Materials and methods

### Apiculture and comb construction

Data sets were obtained from *A. mellifera* hives that were cultivated in an urban setting in Cambridge, Massachusetts between February and October 2020. Once the colonies established a stable population, experimental environments, each consisting of an acrylic cube measuring either 15 cm × 15 cm × 15 cm or 27 cm × 27 cm × 27 cm, were incorporated into their existing commercially available Langstroth hives (Fig. 2a). Substrates for freeform comb building consisted of acrylic or stainless-steel crossbars, 3D-printed substrates, or a combination of both. The acrylic experimental cubes were designed to be easily removable and compatible with downstream micro-CT scanning, without the generation of imaging artifacts, and allowed for control over the initial conditions and modulation of the comb-building environment over time.

A beeswax foundation (hexagonally patterned wax sheets acquired from commercial sources) was applied to the internal crossbar of each of the three experimental cubes (Hives 1–3) to encourage comb building, and the hive’s queen was temporarily placed within a cage in the experimental cube to encourage worker bee migration into these new spaces. Once the colony began building comb within each of the acrylic cubes, the queen was then removed to minimize the production of brood cells and returned to the lower Langstroth hive.

Periodically, during the hive’s comb-building process, each of the experimental cubes was manually rotated so that a different side formed the base. These rotations induced directional changes in the comb growth, similar to those observed in Huber’s experiments^8^. Rotations were conducted every three weeks over a period of three months, and each rotation consisted of a 90-degree rotation along with one of the hive’s horizontal axes. Rather than following a specific predetermined order, the direction of rotation was chosen to place the side of the hive with the least comb volume in the downwards orientation so as to encourage comb building in this direction.

While it would have been useful to reconstruct an interactive 3D volume at every stage of the rotation process for a detailed time-resolved analysis, doing so in a manner that does not further influence the hive’s behavior is a nontrivial task. Any instance of opening the hive to record real-time 3D data or the introduction of additional light sources for photogrammetry purposes would introduce additional variables that could have impacted bee behavior and subsequently comb architecture. Therefore, the methods outlined here were specifically developed to provide a thorough analysis of fully formed comb architectures, and as such, are equally well suited for the investigation of both experimental and wild hives.

### Digitization workflow

After comb construction, the experimental cubes were removed and scanned with an XRA-002 X-Tek µCT imaging system (Nikon Metrology, Tring, UK). Micro-CT scanning was selected both for its ability to provide high-resolution volumetric data, as well as the fact that the depth and angle of comb cells coupled with a highly curved global geometry created many hidden regions that could not be accurately mapped by photogrammetry or laser scanning. The scans were acquired at 70 kV and 200 μA with an exposure time of 1 s and a 0.1° rotational increment over 360°. Reconstructions were performed with density values normalized between 0.0 and 1.0 from 16-bit depth TIFF image slices (Fig. 2c). Volumetric reconstructions (Fig. 2d) were performed using VGStudio Max 3.0 (Volume Graphics, Heidelberg, Germany), and their corresponding image stacks were exported for further analysis. These slices were recombined into volumes of 2000^3^ voxels (eight teravoxels) of ~100 μm × 100 μm × 100 μm and stored as OpenVDB files^36^ for processing and analysis. This resolution was approximately one-fifth the thickness of an average cell wall, which ranged from 500–700 μm. As calculations at such a high resolution can become unwieldy, this resolution was halved for more computationally intensive methods (see Geodesic Distance and Gaussian Curvature tools). Although for the results presented in the present study, we used the 3D modeling software Houdini (SideFX, Ontario, Canada) for the data processing and visualizations, a method for analyzing these volumes using the open-source software 3D Slicer is also provided in the Supplemental Information and is outlined in Fig. S1. We have also made the data sets described in this paper publicly available in a format that can be reconstructed using 3D Slicer, and each of these samples is shown in Fig. S2.

### Reporting summary

Further information on research design is available in the Nature Research Reporting Summary linked to this article.

## Supplementary information

Supplemental Materials

Reporting Summary

## Data Availability

Data generated during the study including micro-CT scans are available in a public repository^37^: 10.5061/dryad.bnzs7h4cm.

## Code availability

Free, open-source software capable of reconstructing the provided data as volumetric images can be found in the README document in the public repository^37^: 10.5061/dryad.bnzs7h4cm. Additional code may be made available upon request. All inquiries should be directed to the corresponding author.
