# Author Correction: Computational methods for the characterization of *Apis mellifera* comb architecture

**DOI:** 10.1038/s42003-022-03827-6

**Published:** 2022-08-22

**Authors:** Christoph Bader, João Costa, Nic Lee, Rachel Smith, Ren Ri, James C. Weaver, Neri Oxman

**Affiliations:** 1 MIT Media Lab, Massachusetts Institute of Technology, Cambridge, MA 02139 USA; 2 Wyss Institute for Biologically Inspired Engineering, Harvard University, Boston, MA 02115 USA

**Keywords:** Structural biology, Computational biology and bioinformatics

Correction to: *Communications Biology* 10.1038/s42003-022-03328-6, published online 16 May 2022.

In the original version of the Article, the second paragraph incorrectly stated “Comb cells are built at an average angle of 13 degrees from an interface where the basal sides of two cells meet^12^, and it has been proposed that this angle helps better retain honey within the cell interiors.” The text should read, “Comb cells are built at an average angle of 13 degrees from an interface where the basal sides of two cells meet, and while it has been proposed that this angle helps better retain honey within the cell interiors, recent evidence suggest that it may also provide additional structural reinforcement for the growing comb^12^.”

This has now been corrected in the PDF and HTML versions of the Article.

