# Supplementary material for: Computational methods for the characterization of Apis mellifera comb architecture
Source: Commun Biol. 2022 May 16;5:468. doi: 10.1038/s42003-022-03328-6 (PMC9110387; doi:10.1038/s42003-022-03328-6)
Supplement: Supplementary file 1 — Supplemental Materials [file 42003_2022_3328_MOESM1_ESM.pdf]

Computational Methods for the Characterization of *Apis mellifera* Comb Architecture Supplemental Information

Christoph Bader[a1], João Costa[a1], Nic Lee[a1], Rachel Smith[a1], Ren Ri[a], James C. Weaver[b], Neri Oxman[a1*]

1 Indicates that these authors contributed equally to this publication

a Media Lab, Massachusetts Institute of Technology, Cambridge Massachusetts, USA.

b School of Engineering and Applied Sciences, Harvard University, Cambridge Massachusetts, USA.

*Corresponding author

Open-Source Software for Comb Reconstruction

The methods described in the present study can also be implemented using free, open-source software, such as 3D Slicer (Fedorov, 2012), which is often employed for the analysis of medical imaging data sets. To demonstrate this point, we imported our CT data into 3D Slicer and defined a region of interest (ROI) by cropping the boundaries defined by the acrylic cube. Using a standard segmentation approach in which we specified electron density thresholds, we were able to separate the three main material components of our data set: honey, wax and the 3D printed parts (figure S1). The resulting 3D model provides enough information for a quick assessment of this particular model showing the spatial distribution of the different materials.

[FIGURE S1]

Publicly Available Datasets and Descriptions

We have also made the CT scan datasets shown here available to the public in the form of image stacks stored in an open repository. Each hive was cultivated in Cambridge, Massachusetts between the months of February and October, 2020. Colonies were provided with supplemental nutrition in the form of sugar syrup and synthetic pollen during the Fall in order to encourage comb production. All acrylic cubes were placed atop established colonies housed within 1 to 2 brood chambers and 1 to 2 supers and covered with an external box to create a dark, enclosed environment. Queens were temporarily moved to the acrylic cubes to encourage integration with the hive. After an acrylic cube was accepted by the hive, the queen was returned to the brood chambers and prevented from entering the acrylic cube through the use of a queen excluder. Descriptions of each hive are included below.

Hives – H 1-3

Hives H1-H3 were experimental replicates, and each were cultivated in a 27 cm x 27 cm x 27 cm acrylic cube with an acrylic crossbar spanning two axes. Wax foundation was attached to each of the acrylic crossbars with metal screws. Supplemental wax was placed at the hive's entrance, and each acrylic cube was periodically rotated to induce perturbations in comb morphology due to changes in the perceived orientation of the external gravitational field.

Hive F – Fracture

Hive F was cultivated in a 15 cm x 15 cm x 15 cm acrylic cube with a 3D printed, highly curved substrate serving as the roof of the cube. The 3D-printed substrate was fabricated from Vero-Clear (RGD-810) resin using a Stratasys J750 3D printer and was coated with melted beeswax prior to placement in the hive. During comb construction, the central region of wax became fractured and the two surfaces became physically separated from one another, which were later repaired by the hive through the joining of a section built from the cube's base with the previous build extending from the 3D printed substrate. *Hive F* contained no metal parts, and as such, may be better suited for material-specific density analysis.

Wild Comb Samples – WC 1-4

In addition to the cultivated hives discussed in this publication, we have included several micro-CT data sets of comb constructed by wild or feral honeybees. These samples were obtained by Super Bee Rescue in Santa Barbara, California when the colonies that constructed them were relocated. All four samples were collected from various hives built within hollow trees.

[FIGURE S2]

[FIGURE S3]

Supplemental Figures

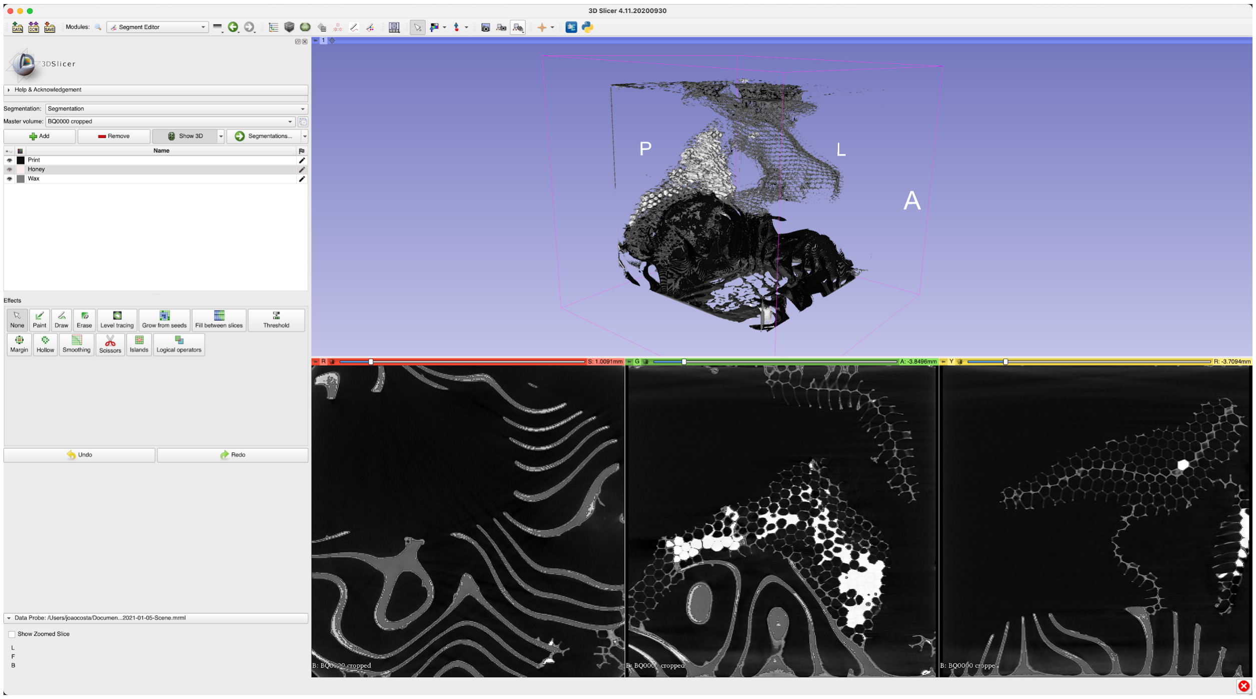

Figure S1 – **Examination of CT Scanned Comb Using Open Source Software.** A screen capture from the publicly available data set of *Hive F,* visualized using the open-source software, 3D Slicer. CT scans can be reconstructed in 3D Slicer and the measured electron density values can be used to segment specific regions of the scanned comb, such as wax, honey, or the 3D-printed substrate.

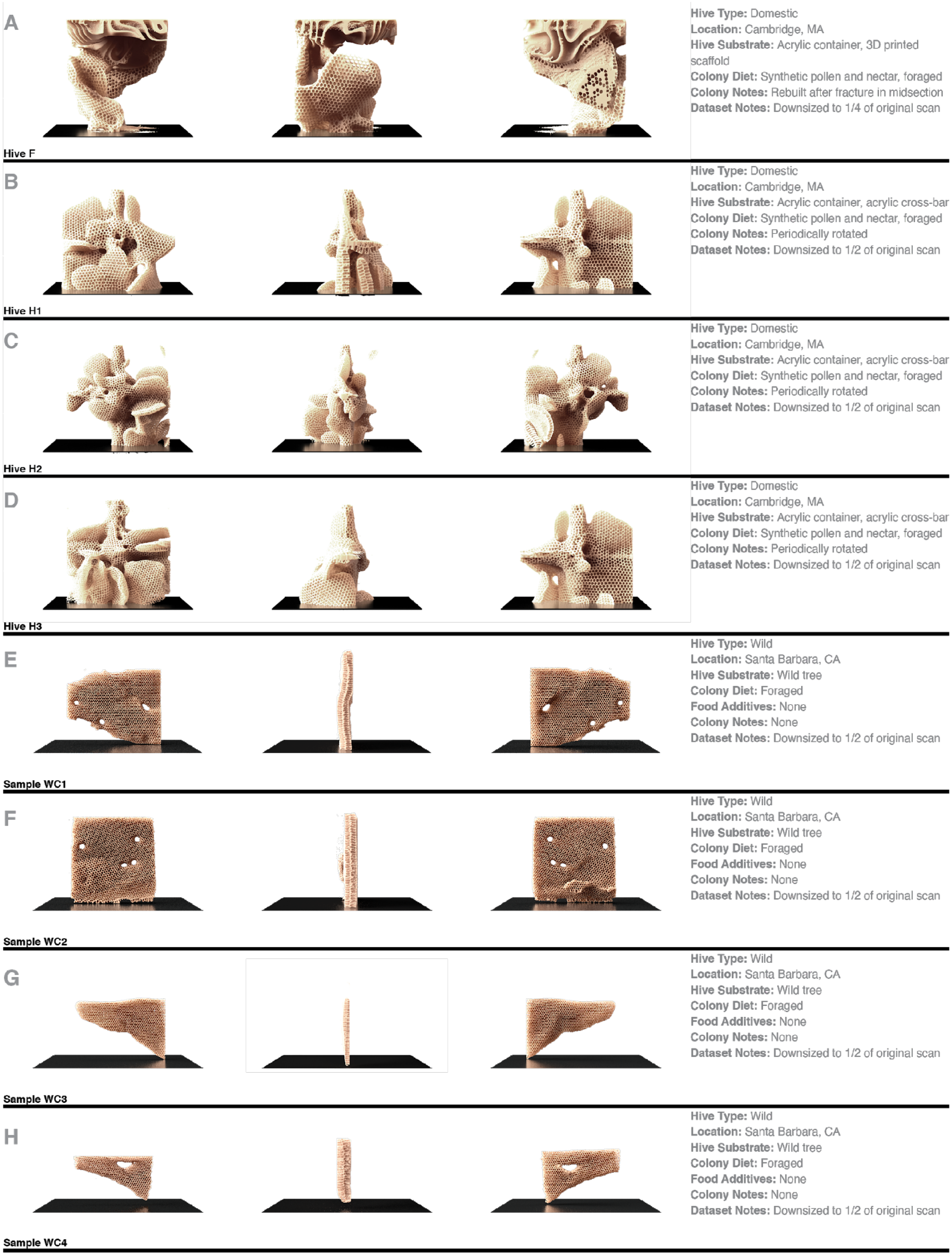

Figure S2 – **Reconstructions of Publicly Available Comb micro-CT data sets.** A volumetric reconstruction of each hive is displayed from three different viewing angles. *Hives 1-3* (**A-C**) were periodically rotated during hive construction. *Hive F* was constructed upon a 3D printed substrate, fractured during construction, and was subsequently repaired by the colony (**D**). Samples WC 1 (**E**), WC 2 (**F**), WC 3 (**G**), and WC 4 (**H**) were obtained from wild or feral hives.

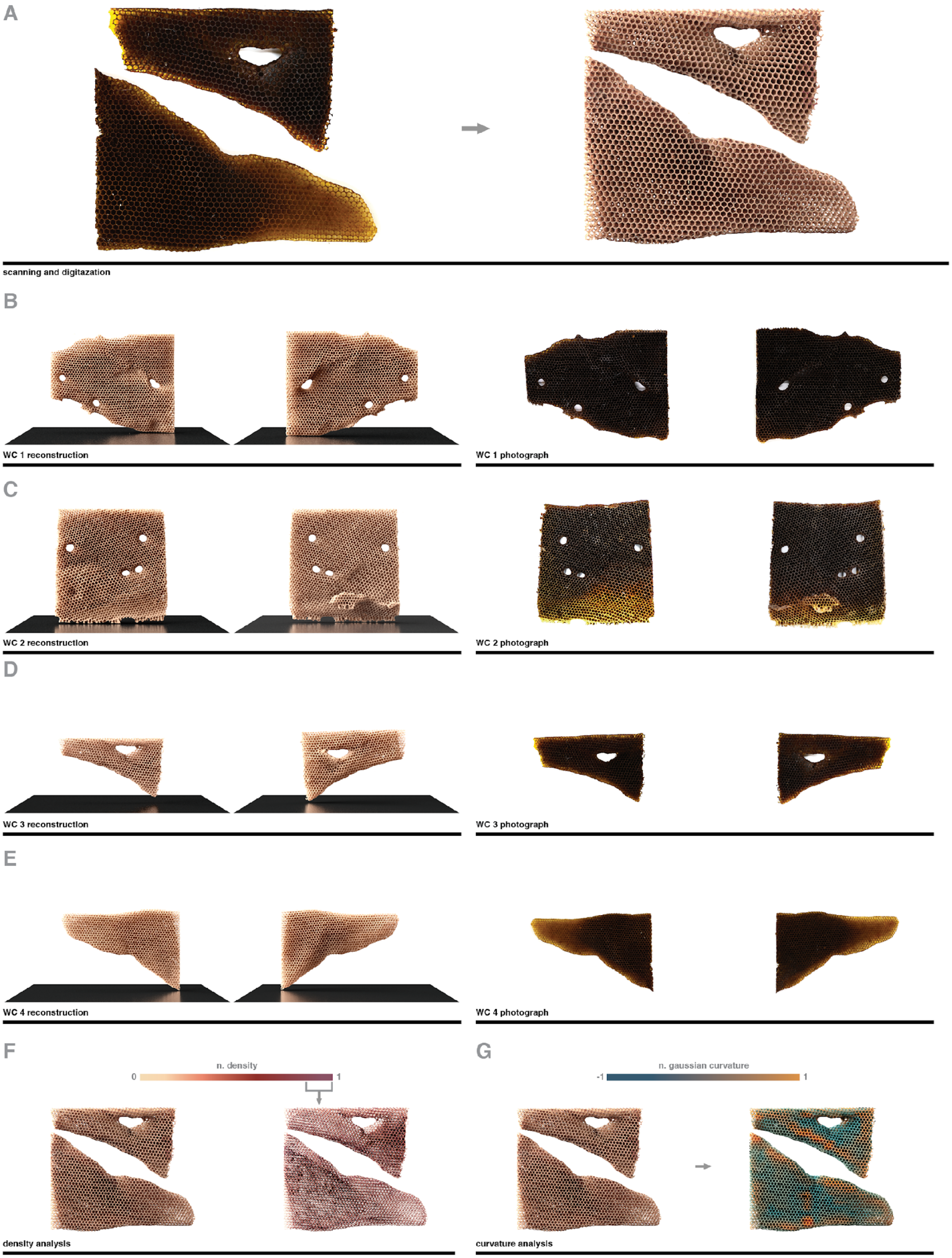

Figure S3 – **Wild Comb Samples.** The four samples of wild comb contained in the WC data set were archived as segmented volumetric reconstructions. Samples WC 1(**B**) WC 2 (**C**), WC 3 (**D**), and WC 4 (**E**) are shown alongside photographs of the original samples. These data sets are suitable for analysis using the methods outlined in this study including density analysis (**F**) and Gaussian curvature analysis (**G**).
