# Supplementary material for: Computational methods for the characterization of Apis mellifera comb architecture
Source: Commun Biol. 2022 May 16;5:468. doi: 10.1038/s42003-022-03328-6 (PMC9110387; doi:10.1038/s42003-022-03328-6)
Supplement: Supplementary file 2 — Reporting Summary [file 42003_2022_3328_MOESM2_ESM.pdf]

nature research

Corresponding author(s): *Double-blind peer review submissions: write DBPR and your manuscript number here instead of author names.*

Last updated by author(s): *YYYY-MM-DD*

Reporting Summary

Nature Research wishes to improve the reproducibility of the work that we publish. This form provides structure for consistency and transparency in reporting. For further information on Nature Research policies, see our Editorial Policies and the Editorial Policy Checklist.

Statistics

For all statistical analyses, confirm that the following items are present in the figure legend, table legend, main text, or Methods section.

n/a	Confirmed	
[ ]	[x]	The exact sample size (n) for each experimental group/condition, given as a discrete number and unit of measurement
[ ]	[x]	A statement on whether measurements were taken from distinct samples or whether the same sample was measured repeatedly
[x]	[ ]	The statistical test(s) used AND whether they are one- or two-sided<br>*Only common tests should be described solely by name; describe more complex techniques in the Methods section.*
[ ]	[x]	A description of all covariates tested
[ ]	[x]	A description of any assumptions or corrections, such as tests of normality and adjustment for multiple comparisons
[ ]	[x]	A full description of the statistical parameters including central tendency (e.g. means) or other basic estimates (e.g. regression coefficient) AND variation (e.g. standard deviation) or associated estimates of uncertainty (e.g. confidence intervals)
[x]	[ ]	For null hypothesis testing, the test statistic (e.g. F, t, r) with confidence intervals, effect sizes, degrees of freedom and P value noted<br>*Give P values as exact values whenever suitable.*
[x]	[ ]	For Bayesian analysis, information on the choice of priors and Markov chain Monte Carlo settings
[x]	[ ]	For hierarchical and complex designs, identification of the appropriate level for tests and full reporting of outcomes
[x]	[ ]	Estimates of effect sizes (e.g. Cohen's d, Pearson's r), indicating how they were calculated

Our web collection on statistics for biologists contains articles on many of the points above.

Software and code

Policy information about availability of computer code

Data collection	The supplement provides a description of how to view our publicly accessible data using the free open-source software 3D Slicer. Analysis and visualizations provided in this manuscript were conducted using Houdini (Side FX) software version 18.
Data analysis	Analysis and visualizations provided in this manuscript were conducted using Houdini (Side FX) software version 18.

For manuscripts utilizing custom algorithms or software that are central to the research but not yet described in published literature, software must be made available to editors and reviewers. We strongly encourage code deposition in a community repository (e.g. GitHub). See the Nature Research guidelines for submitting code & software for further information.

Data

Policy information about availability of data

All manuscripts must include a data availability statement. This statement should provide the following information, where applicable:

- Accession codes, unique identifiers, or web links for publicly available datasets
- A list of figures that have associated raw data
- A description of any restrictions on data availability

All data analyzed for this manuscript including micro-CT image stacks are compiled in a public repository at https://github.com/MediatedMatter/Comb-Scan-Database/tree/master/Image_Stacks.

Field-specific reporting

Please select the one below that is the best fit for your research. If you are not sure, read the appropriate sections before making your selection.

[x] Life sciences [ ] Behavioural & social sciences [ ] Ecological, evolutionary & environmental sciences

For a reference copy of the document with all sections, see nature.com/documents/nr-reporting-summary-flat.pdf

Life sciences study design

All studies must disclose on these points even when the disclosure is negative.

Sample size	This study focuses on novel methods for the characterization of comb morphology and is intended to lay the groundwork for further analysis in for work with a model organism where data collection is historically challenging. Four domestic hives were included in the analysis to ensure consistency of the methodological outcomes, and the manuscript does not use statistical methods to draw specific conclusions.
Data exclusions	No data was excluded from the manuscript.
Replication	Modifications to hive environments such as rotation or the insertion of scaffolds were conducted on four hives simultaneously. The described analysis methods were applied to each of these hives to ensure consistency.
Randomization	Randomization was not required for this study as direct conclusions were drawn from statistical methods. Rather, the study focuses on novel high-throughput methods for the characterization of honey-comb morphology and contrasts the outputs of these methods against previously reported empirical data.
Blinding	Blinding was not required for this data as direct conclusions were drawn from statistical methods. However, the described methods were conducted on four domestic honeybee hives to ensure consistency and compared against samples of wild comb from colonies in another state. The methods generated consistent results across these samples in allowing for the observation of material distributions and morphological patterns in constructed honeycomb.

Reporting for specific materials, systems and methods

We require information from authors about some types of materials, experimental systems and methods used in many studies. Here, indicate whether each material, system or method listed is relevant to your study. If you are not sure if a list item applies to your research, read the appropriate section before selecting a response.

Materials & experimental systems

n/a	Involved in the study	
[x]	[ ]	Antibodies
[x]	[ ]	Eukaryotic cell lines
[x]	[ ]	Palaeontology and archaeology
[ ]	[x]	Animals and other organisms
[x]	[ ]	Human research participants
[x]	[ ]	Clinical data
[x]	[ ]	Dual use research of concern

Methods

n/a	Involved in the study	
[x]	[ ]	ChIP-seq
[x]	[ ]	Flow cytometry
[x]	[ ]	MRI-based neuroimaging

Animals and other organisms

Policy information about studies involving animals; ARRIVE guidelines recommended for reporting animal research

Laboratory animals	Apis mellifera colonies (domestic).
Wild animals	Apis mellifera colonies (feral) provided comb analyzed in these studies. The comb was collected by Super Bee Rescue in California after colonies were relocated due to interference with human activities.
Field-collected samples	Domestic hives were kept on a rooftop in Cambridge, Massachusetts and tended to by the research team. Colonies were fed supplemental nectar and pollen weekly and monitored for parasites. Honey was not collected from hives to ensure adequate nutrition.
Ethics oversight	No ethical approval was required for this study as it did not involve human researchers or methods that diverged substantially from common agricultural practices.

Note that full information on the approval of the study protocol must also be provided in the manuscript.
